# IAPP blocks anti-breast cancer function of CD8^+^T cells via targeting cuproptosis

**DOI:** 10.3389/fimmu.2024.1481129

**Published:** 2024-11-25

**Authors:** Dandan Guo, Zhijian Huang, Qianqian Wang, Wei Chen, Yu Huang, Xinhao Sun, Jian Chen, Shuying Feng

**Affiliations:** ^1^ Medical College, Henan University of Chinese Medicine, Zhengzhou, China; ^2^ Henan Engineering Research Center for Chinese Medicine Foods for Special Medical Purpose, Zhengzhou, China; ^3^ Department of Breast Surgical Oncology, Clinical Oncology School of Fujian Medical University, Fujian Cancer Hospital, Fuzhou, China; ^4^ Department of Thyroid and Breast Surgery, Ningde Municipal Hospital of Ningde Normal University, Ningde, China

**Keywords:** breast cancer, IAPP, cuprotosis, biomarkers, ICR-DEGs

## Abstract

**Background:**

Breast cancer (BRCA) is the most prevalent type of cancer worldwide. As a highly heterogeneous cancer, it has a high recurrence rate. Since its biological behavior can be regulated by immunity and cuprotosis, so exploring potential therapeutic target to mediate immunity and cuprotosis is of great significance for BRCA therapy.

**Methods:**

The immune-related genes and immune-cuprotosis-related deferentially expressed genes (ICR-DEGs) were identified by mining the TCGA database. Prognostic analysis, differential expression analysis, univariate and lasso regression analyses were used to determine their independent prognostic values. To evaluate the relationship between ICR-DEGs and immune scores, we constructed a prognostic risk model to evaluate immune checkpoints, and then the role of tumor immune microenvironment in BRCA was explored. Furthermore, anti-BRCA function and mechanism of islet amyloid poly-peptide (IAPP) mediated CD8^+^T cells were verified by means of flow cytometry, ELISA, and subcutaneous transplantation tumor model.

**Results:**

All results suggested that immune-cuprotosis-related genes were a potential predictor of BRCA’s response to immune checkpoint inhibitors and immunotherapy biomarkers. Thereby downregulation of IAPP reduced cuprotosis of CD8^+^T or Her2**-**CAR-T cells to promote the anti-BRCA function both *in vitro* and *in vivo*.

**Conclusion:**

Our research had clarified the function and mechanism of IAPP in CD8^+^T cells, providing new ideas for improving the diagnosis and treatment of BRCA.

## Introduction

Breast cancer (BRCA) has overtaken lung cancer and was regarded as the major factor affecting women’s health in mortality worldwide (1–3). Factors such as infections, unhealthy diet, occupational hazards, smoking, genetic predisposition, and environmental pollution, contribute to the development of cancer (4). Given the high heterogeneity of BRCA, identification of biomarkers that can aid in diagnosing, assessing disease progression, and predicting outcomes is essential for timely recognition and disease management during treatment (5).

Cuprotosis, a newly discovered mode of regulated cell death, differs significantly from pyroptosis, ferroptosis, and apoptosis in its characteristics. Normally, intracellular copper concentration is maintained at extremely low levels in cancer cells. Cu^2+^ is catalyzed by STEAP proteins on the membrane surface to be reduced to Cu^+^, resulting in stronger cytotoxicity (6–8). The copper ions enter into the cell through the outer and inner membrane of mitochondria through COX17 and SLC25A3, and finally enter the mitochondrial matrix. The imbalance of copper homeostasis can lead to cellular metabolic disorders. When copper ions accumulate excessively due to ion carriers or transport proteins, on the one hand, FDX1 reduces Cu^2+^ to more toxic Cu^+^, inhibiting the synthesis of mitochondrial respiratory-related Fe-S cluster protein, causing protein toxicity stress response and ultimately leading to cell death. On the other hand, FDX1, as an upstream regulatory factor for protein thioacylation modification, participates in regulating the thioacylation of DLAT. Cu^2+^ can directly bind and induce the hetero-polymerization of DLAT, and subsequently leads to protein toxicity stress and induces cell death (9–12). Although the relationship between cell death and immunity has been found in many studies, such as ferroptosis (13, 14), autophagy (15, 16), and apoptosis (17, 18), the mechanism behind cuproptosis and immunity in BRCA is yet to be clarified.

To investigate the differential expression of genes associated with immune cuprotosis, data of BRCA related gene expression was retrieved from the cancer genome atlas (TCGA) database. A model that effectively incorporates multiple genes for predicting the survival of BRCA patients was established, and correlation between risk scoring model and immune status was determined to provide a diagnostic basis for treatment and to uncover novel therapeutic targets. Our data demonstrated that islet amyloid polypeptide (IAPP) could serve as a novel biomarker for diagnostic and treatment of BRCA. This study explored the function and mechanism of IAPP in CD8^+^T cells, and the data showed that downregulation of IAPP could enhance the killing function of CD8^+^T or CAR-T cells by reducing the copper concentration, provides a new idea for improving the diagnosis and treatment of BRCA.

## Materials and methods

### Screening of immune cuprotosis-related DEGs

Data from the TCGA database were downloaded to compare deferentially expressed genes (DEGs) associated with immune cuprotosis in BRCA patients. Totally 17,500 human immunity-related genes (IRGs) and 1,956 cuprotosis-related genes (CRGs) were downloaded from the GeneCard database (https://www.genecards.org/) and the ImmPort database (https://www.immport.org./home). The cutoff condition was set to logFC≥2 with *P*<0.05.

### Establishment and validation of prognosis model

Following the DEGs screening, significant prognostic immune cuprotosis-related DEGs (ICR-DEGs) with a *P*<0.05 were identified through univariate Cox analysis of overall survival (OS). The predictive model for these ICR-DEGs was established using the least absolute shrinkage and selection operator (LASSO) proportional regression. A total of 1,089 BRCA patients were randomly assigned in a 1:1 ratio to either training cohort or test cohort with the purpose of developing and validating risk scores. Specifically, 545 patients comprised the training cohort, while 544 patients constituted the test cohort. For the training cohort, a linear combination of expression values for each prognostic gene was utilized to formulate our prognostic risk profile specific to the immunocuprotosis-related signature (ICRSig). The median risk score served as the threshold in the TCGA-BRCA dataset to divide patients into low-risk and high-risk groups. The distribution characteristics of different groups were analyzed by principal component analysis (PCA) and R-package analysis. Finally, effectiveness of prognostic indicators was assessed by the area under the curve of “time receiver operating characteristic curve”.

### Survival analysis and verification

The ClusterProfiler software package was utilized to conduct functional enrichment analysis of ICR-DEGs, aiming to investigate their functional annotations and enriched pathways. To further assess the expression and prognostic significance of ICR-DEGs in BRCA, differential expression and prognostic analyses were conducted using the “survival” software package. These analyses were guided by the risk ratio (HR) derived from the Cox proportional hazards model and the Kaplan-Meier model.

### Correlation analysis of clinical pathology and construction of histogram

We conducted an analysis of association between ICR-DEGs and clinical pathological features by utilizing the “survival” package in R along with clinical pathological attributes. Utilizing the R package “rms”, we obtained the column alignment and calibration curve. Additionally, risk scores related to the prognostic model were utilized as prognostic factors for assessing 1-, 3-, and 5-year OS.

### Relationship between risk score and immune cell infiltration

The ssGSEA and CIBERSORT R scripts were used for quantification of the relative ratios of infiltrating immune cells. Spearman’s rank correlation analysis was employed to investigate the correlation between risk rating values and immunological infiltrating cells. Furthermore, we assessed two distinct mechanisms of tumor immune escape using the TIDE algorithm, leveraging the ICR-DEG markers.

### T cell isolation and flow cytometry analysis

Human peripheral T cells were purified using anti-human CD3 microbeads (Miltenyi). The sorted T cells were activated with anti-CD3/CD28 microbeads (Miltenyi) at a ratio of 1:1 for 48 h with 100 IU/mL IL-2. Activated T cells were infected with the lentivirus which packaged with pll3.7 plasmid including mU6 promoter to expressing shRNA and EF-1α to expressing EGFP. And then, the infected T cells were expanded for another 12 days with fresh media and IL-2 supplementation every 2 days. For preparation of CAR-T cells, T cells were seeded into 24-well plates and transduced with lentivirus which packaged with pll3.7 plasmid including mU6 promoter to expressing shRNA and EF-1α to expressing Her2-scfv at an MOI of 10. Consequently, treated T cells were collected by centrifugation for 2 h at 1800 rpm/min. 1×10^6^ single suspension cells were stained with the proper antibodies diluted in cell staining buffer (420201, BioLegend, USA) for 30 min at 4°C. Anti-Human CD45-FITC (Biolegend, 561865), anti-Human CD3-Alexa Fluor^®^ 488 (Biolegend, 557694), anti-Human CD4-APC (Biolegend, 561841), and anti-Human CD8-APC-Cy7 (Biolegend, 348813) was used separately for surface staining. Anti-human IFN-γ-Percp-cy5.5 (Invitrogen, 45-7319-42), anti-human, Granzyme B-PE (Invitrogen, 12-8899-41), anti-human and TNF-α-BV786 (Biolegend, 569461) were used separately for intracellular staining.

### Copper detection

As reported previously, ICP-MS is used for detecting copper by Keystone analytics (19). Briefly, cells were spiked with 50 μL of indium (100 ng/mL) as an internal standard and thoroughly mixed before being pumped into an ICP-MS. Multi-element standard solutions containing Cu and a working internal solution of indium were prepared from individual element standard stock solutions sourced from LGC (Manchester, NH). An Agilent 7800 inductively coupled plasma mass spectrometer (located in Santa Clara, CA) was utilized to measure the Cu levels.

### Detection of cuprotosis protein marker

As reported previously, proteins isolated from CD8^+^T cells were separated on 10% SDS-PAGEs and transferred onto polyvinylidene difuoride (PVDF) membranes (Millipore, Bedford, MA, USA). After blocking with 5% skim milk, membranes were incubated with the primary antibodies at 4°C overnight, and then incubated with HRP-labeled goat anti-rabbit antibody for 0.5 h. Finally, the immunoreactive signals were detected using the ECL detection system (SuperSignal West Femto Maximum Sensitivity Substrate, Termo Fisher Scientifc, IL, USA). The following primary antibodies were applied: rabbit anti-FDX1 (ab108257, Abcam, USA), rabbit anti-SLC31A1 (ab133385, Abcam, USA), and rabbit anti-Actin (ab8227, Abcam, USA). Besides, GzmB, IFN-γ, and TNF-α concentrations were measured using ELISA kits from Biolegend (USA)/R&D systems (USA) as manufacturers’ protocols.

### Animal model

All animal studies were carried out under protocols approved by the Institutional Animal Care and Use Committee of Henan University of Chinese Medicine (IACUC-202401004). 6 to 8-week-old female NSG mice were used to establish subcutaneous tumor models in this study. A suspension of 10^6^ MDA-MB-231 cells was subcutaneously injected into the mice as well as the NC CD8^+^T cells, sh-RNA-IAPP (sh-RNA) CD8^+^T cells, and TTM were prepared for treatment of mice. When tumor volume reached approximately 80 mm^3^, mice were randomly divided into four groups: NC, NC+TTM, sh-RNA, sh-RNA+TTM (n=5). Preparation of NC and sh-RNA CD8^+^T cells, tail vein injection of 5×10^6^ (every 7 days, 3 times in total) combined with oral administration of tetrathiomolybdate (TTM) 0.3mg/d. For CAR-T cells treatment of mice, a suspension of 10^6^ MDA-MB-231 cells was subcutaneously injected into the mice, and then 5×10^6^ CAR-T cells were infused via tail vein. The tumor growth was measured using IVIS.

### Statistical analysis

The data processing and statistical analysis was conducted using Perl (version 5.30.2) and R software (version 4.0.2), respectively. Multivariate Cox regression analysis assessed the prognostic significance, while PCA was executed utilizing the ggplot2 package in R. The Kaplan-Meier curve and logrank test were utilized to analyze survival differences between groups. Gene correlation analysis relied on the Pearson correlation coefficient test, while risk scoring and correlation analysis of immune cells and genes utilized the Spearman correlation coefficient test. Experimental data were displayed using Prism v7.0 (GraphPad Software), with mean ± SD reported and statistical significance tested using Student’s t-test and one- and two-way ANOVA. The logrank test was applied to analyze survival curves, with statistical significance defined as *P*<0.05.

## Results

### Screening of ICR-DEGs in BRCA

**Figure 1:**
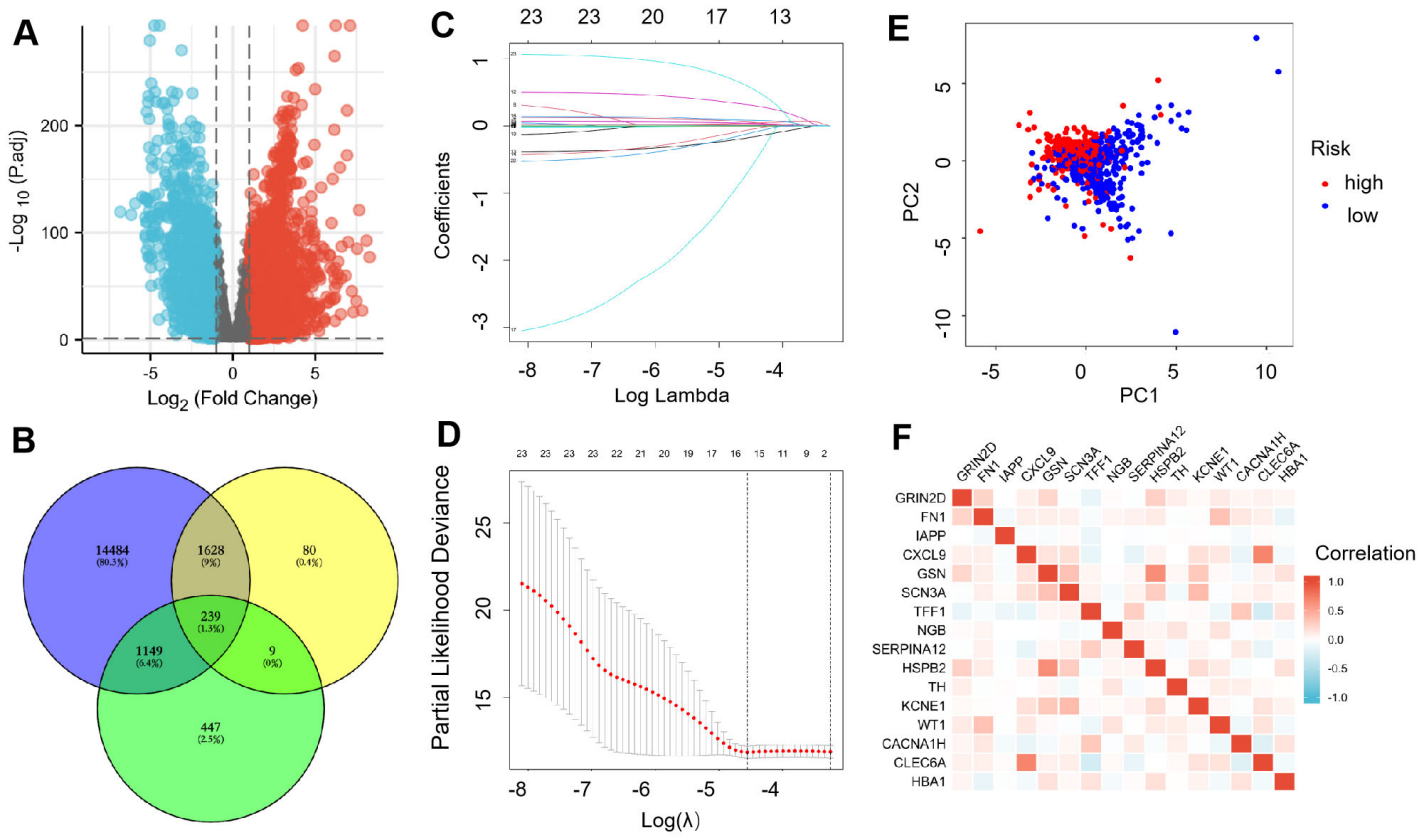
Expression of ICR-DGEs in BRCA tissue and normal breast tissue. **(A)** DEGs volcano diagram of BRCA and adjacent tissues (Red represents up-regulated genes, and blue represents down-regulated genes). **(B)** Venn diagram of 239 immune-cuprotosis genes. **(C)** LASSO regression analysis of 239 DEGs. **(D)** A 10-fold cross-validation was used for calculating the optimal lambda, which results in a minimum mean cross-validation error (16 ICR-DEGs were used in LASSO model). **(E)** PCA map of the high-risk and the low-risk groups. **(F)** The gene related network diagram of prognosis model.

We downloaded the genes from TCGA database and found that 9031 up-regulated DEGs and 5591 down-regulated DEGs (**Figure 1A**). After that, DEGs, IRGs, and cuprotosis-related genes were analyzed together. The Venn diagram showed 239 co-expressed genes were observed among the three sections (**Figure 1B**). Lasso regression was used to establish a predictive model of risk associated with DEGs. 16 genes (GRIN2D, FN1, IAPP, CXCL9, GSN, SCN3A, TFF1, NGB, SERPINA12, HSPB2, TH, KCNE1, WT1, CACNA1H, CLEC6A, and HBA1) were utilized to build the risk signature, meanwhile, the risk score was calculated according to the coefficients from LASSO algorithm (**Figures 1C, D**). PCA results validated differential expression between high-risk and low-risk groups divided according to median risk score (50%) in patients with BRCA (**Figure 1E**). Heatmap showed the expression relation of different genes (**Figure 1F**).

### Construction and validation of prognosis model

**Figure 2:**
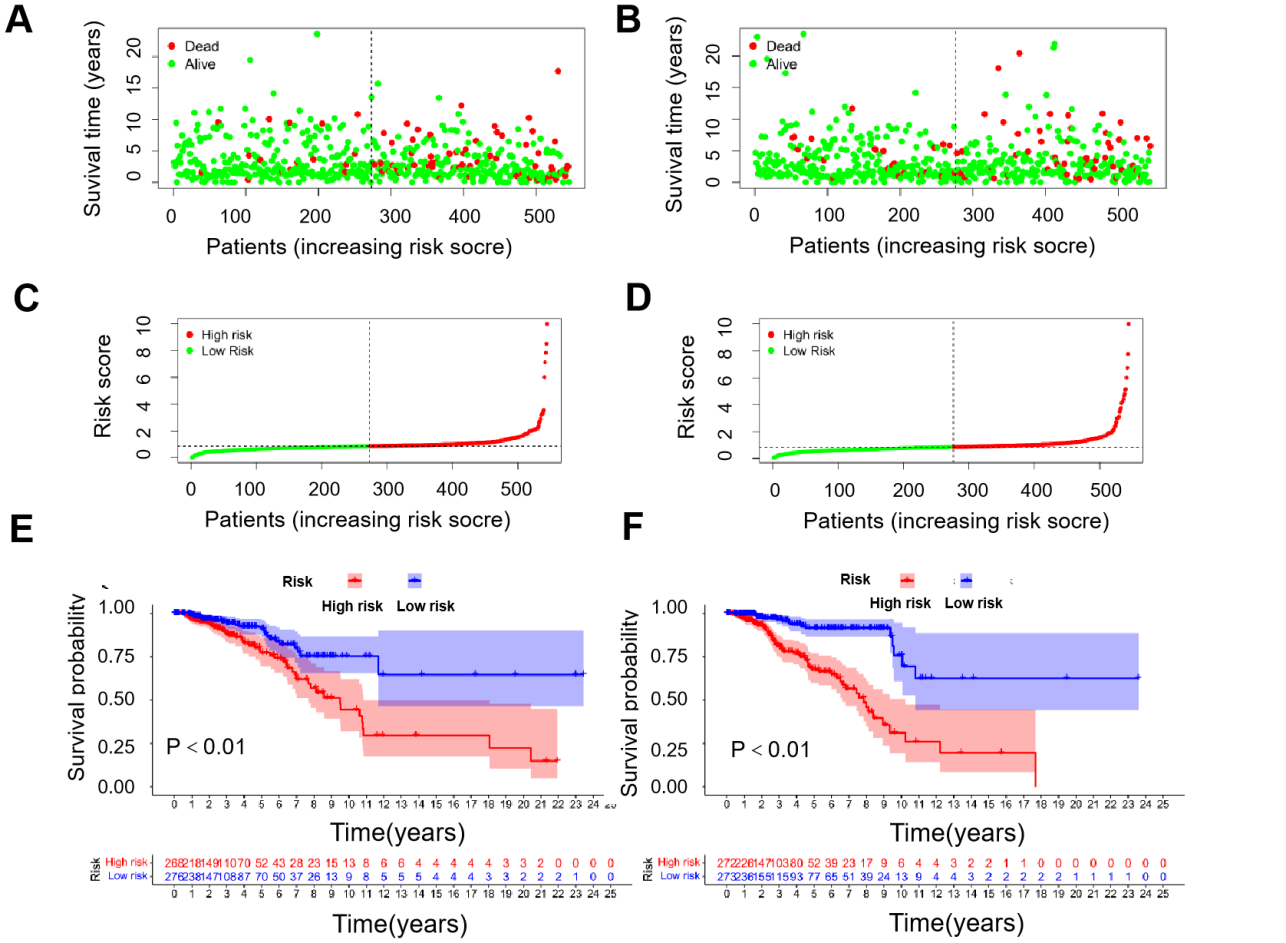
Correlation between prognostic models and OS. **(A)** Survival time and survival status between low- and high-risk groups in the training cohort. **(B)** Survival time and survival status between low- and high-risk groups in the trial cohort. **(C)** Exhibition of immune-cuprotosis-related model based on risk score in the training cohort. **(D)** Exhibition of immune-cuprotosis-related model based on risk score in the trial cohort. **(E)** Kaplan-Meier survival curve of survival probability of patients between low- and high- risk groups in the training cohort. **(F)** Kaplan-Meier survival curve of survival probability of patients between low- and high- risk groups in the trial cohort.

Using the median risk score, patients were stratified into high-risk and low-risk groups, forming training cohorts that were subsequently analyzed (**Figures 2A–D**). Notably, there were statistically significant differences in OS between the two risk groups within the training cohort (**Figure 2E**, *P*<0.01), which was repeated in both trial cohorts (**Figure 2F**, *P*<0.01). The data revealed that patients with lower risk scores exhibited a more favorable clinical prognosis compared to those with higher risk scores, aligning with the findings observed in both groups within each cohort.

### Risk score acts as a prognostic factor

**Figure 3:**
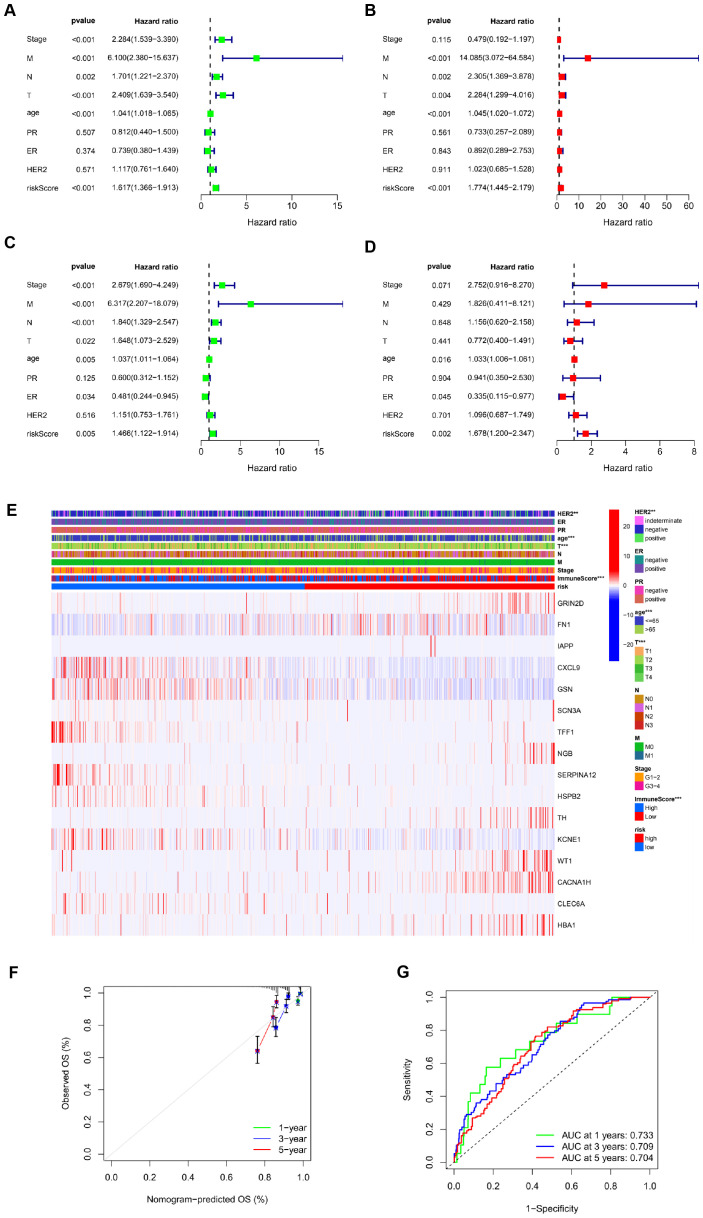
Risk score as an independent prognostic factor. **(A)** Univariate Cox regression results identified in the training cohort. **(B)** Univariate Cox regression results identified in the test cohort. **(C)** Multivariate Cox regression analysis results identified in the training cohort. **(D)** Multivariate Cox regression analysis results identified in the test cohort. **(E)** The heat map utilizing the data of clinical pathological characteristics of patients, (the higher red intensity, the higher expression; the higher blue intensity, the lower expression). **(F)** ROC curves were used to compare the 1-, 3-and 5-year outcomes of histograms in the TCGA cohort. **(G)** Calibration curves for BRCA patients in the TCGA cohort predicting 1-,3-, and 5-year survival (^**^ *P*<0.01, ^***^ *P*<0.001).

The TCGA cohort (serving as the training set) showed a notable correlation between age, risk score, and OS through univariate Cox regression analysis (*P*<0.001, **Figures 3A, B**). Additionally, multivariate Cox regression analysis indicated a significant link between age (*P*<0.001), risk score (*P*<0.001), and OS (**Figures 3C, D**). These results suggest that ICR-DEGs are an independent prognostic factor for BRCA. All results show that ICR-Sig has the ability to accurately predict OS. To investigate which gene expression is associated with prognostic model in clinical characteristics, an expression heatmap was generated based on the correlation of clinical features. The data revealed significant differences in HER2 expression, age, T stage, and immune score between the two risk groups, whereas no significant differences were observed in ER, PR, N stage, M status, and overall stage between the two risk groups (**Figure 3E**). The calibration plot was aligned diagonally, confirming the predictive value of prognostic histogram for OS at 1, 3, and 5 years (**Figure 3F**). The AUC values for bar graphs 1, 3, and 5 years were 0.733, 0.709, and 0.704, separately, demonstrating the good specificity and sensitivity of bar graphs to OS (**Figure 3G**). According to the individual situation, age and TNM were reclassified as prognostic and clinical pathology factors for BRCA patients in the TCGA cohort. Kaplan-Meier survival analysis showed that the survival rate of each subgroup significantly prolonged the survival time of patients regardless of age and TNM stage, except that the high-and low-risk prognosis curve was not significant in the T4 subgroup, indicating that ICRSig had good prediction ability in most subclinical subgroups (**Supplementary Figure S1**). In a word, the histogram constructed by ICRSig has good ability in prognosing patients with BRCA.

### Comprehensive analysis between risk score and TIME

**Figure 4:**
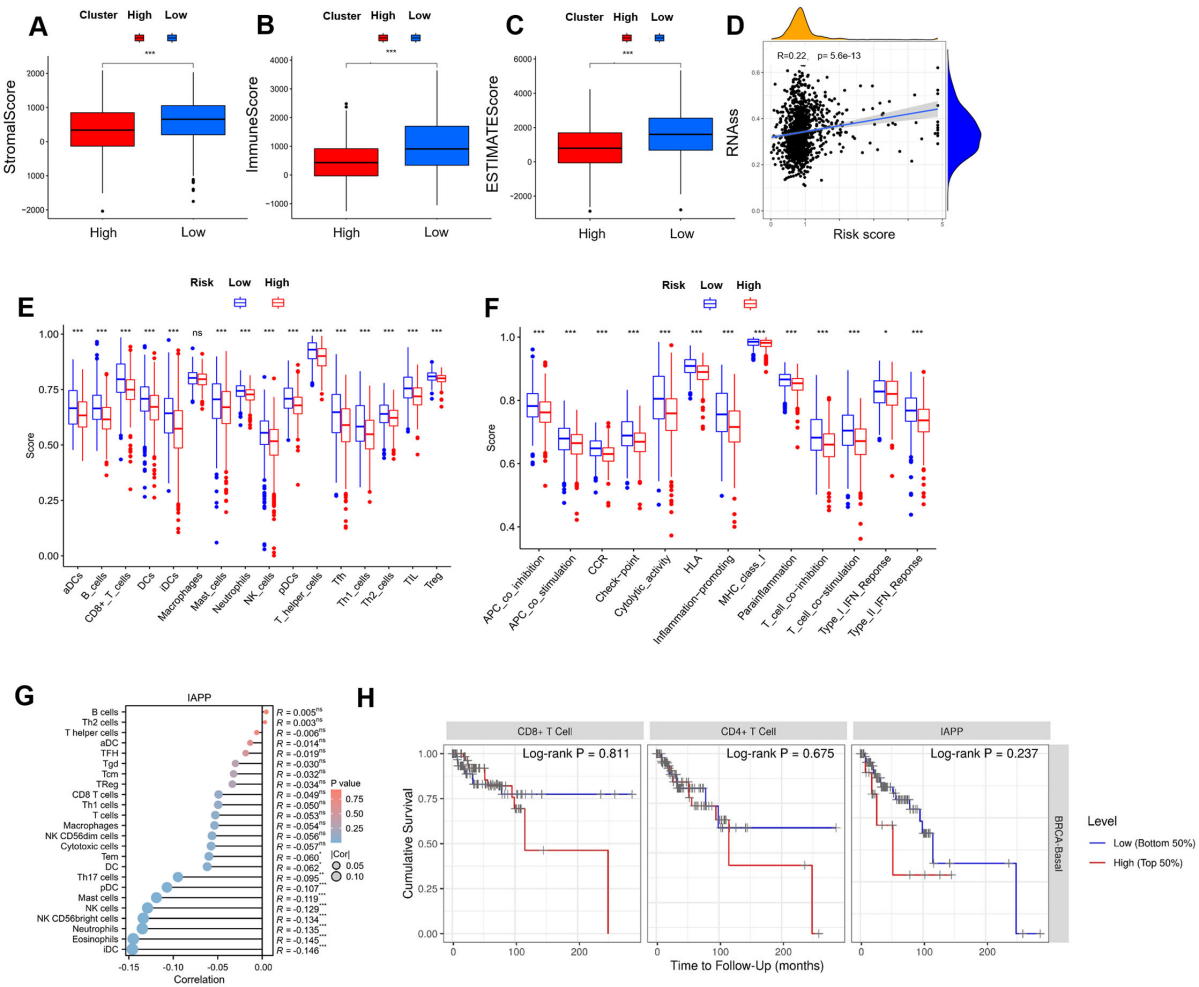
Comprehensive analysis between risk score and TIME. **(A-C)** Comparison of ESTIMATE, Stromal, and Immune scores between the high-risk and low-risk subgroups in the immune microenvironment, immune escape, and immune checkpoint analysis. **(D)** Stemness characteristics. **(E)** A block diagram of ICRSIG and immuno-infiltrating cells. **(F)** A block diagram of ICRSig and immune function. **(G)** The correlation between IAPP and immuno-infiltrating cells. **(H)** The correlation between IAPP and survival (^*^ *P*<0.05, ^**^ *P*<0.01, ^***^ *P*<0.001).

As shown in **Figures 4A–C**, high-risk patients exhibited lower scores in terms of TIME, matrix, immune, and ESTIMATE compared to low-risk patients. For tumor stem cells, we calculated the relation between risk score (R=0.22, *P*=5.6e-13, **Figure 4D**) and stemness scores. For better investigation of the complex crosstalk between ICRSig and immune signals, we assessed the immune infiltrate spectrum of immune cells from BRCA samples. Additionally, we compared the relationship between infiltrated immune cells and ICRSig, observing a marked decline in CD8^+^T cells within the high-risk group (**Figure 4E**, *P*<0.001). CD8^+^T cells decreasing is related to tumor progression and immune suppression. Compared to the low-risk group, the high-risk group showed diminished checkpoint and T-cell co-inhibition function, pointing to stronger immunosuppression in this group (**Figure 4F**, *P*<0.001). To explore the key gene regulating immune function, we re-analyzed the 16 genes and found that IAPP was negatively associated with immune cell infiltration (**Figure 4G**, *P*<0.001 and **Supplementary Figure S2**). Consistent with the previous data, the survival of patients was shorter in the higher expression of IAPP (**Figure 4H**, *P*=0.811, *P*=0.0.675, *P*=0.237). Our research found that IAPP might be a key factor mediating the anti-BRCA effect of CD8^+^T cells via regulating cuproptosis.

### Downregulation of IAPP promotes CD8^+^T cell viability and function

**Figure 5:**
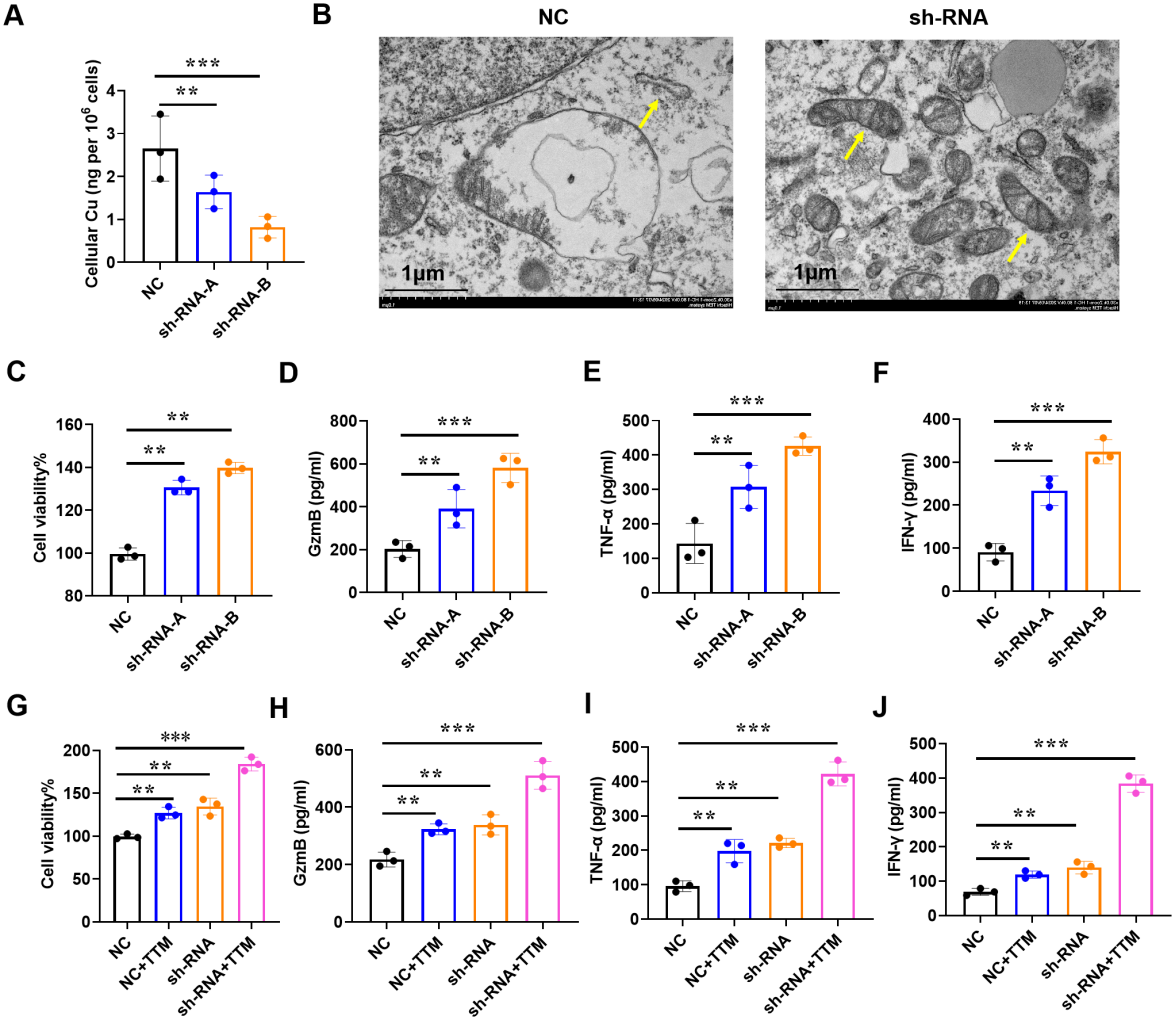
Downregulation of IAPP mediates copper to promote CD8^+^T cell viability and function. **(A)** Detection of copper concentration in IAPP deficiency of CD8^+^T cells (sh-RNA-A and sh-RNA-B) by ICP-MS. **(B)** The morphology of mitochondria was detected by electron microscopy. **(C)** Detection of CD8^+^T cells viability. **(D–F)** Detection of GzmB, TNF-α, IFN-γ secretion in CD8^+^T cells after downregulating IAPP with 1µM Cucl_2_ and 100nM elesclomol, respectively. **(G–J)** Detection of cell viability and GzmB, TNF-α, IFN-γ secretion in CD8^+^T cells with or without 20µM TTM and 1µM Cucl_2_ (Mean ± SD; ^**^ *P*<0.01;^***^ *P*<0.001).

Among tumor-infiltrating lymphocytes, CD8^+^T cells served as a predictors of overall treatment response and survival in BRCA patients. Low infiltration of CD8^+^T cells is linked to tumor progression and immunosuppression (20). Subsequently, we downregulated the expression of IAPP in CD8^+^T cells (**Supplementary Figure S3**, *P*<0.01, *P*<0.001) and observed that it did not affect the cell viability and function of CD8^+^T cells in the absence of CuCl_2_ (**Supplementary Figure S4**). Once in the presence of CuCl_2,_ IAPP deficiency (sh-RNA-A and sh-RNA-B) not only reduced copper concentration (**Figure 5A**, *P*<0.01, *P*<0.001), but also improved the mitochondria damage including mitochondrial morphology and number of ridges (**Figure 5B**). Meanwhile, downregulating IAPP could reduce the expression of FDX1 and SLC31A1, which implied IAPP regulates cuprotosis in CD8^+^T cells (**Supplementary Figure S5**, *P*<0.001). Next, sh-RNA-B were used for further study. Interestingly, viability and secretion of GzmB, TNF-α, and IFN-γ in IAPP down-regulated CD8^+^T cells were obviously increased after treatment with CuCl_2_ (**Figures 5C–F**, *P*<0.01, *P*<0.001). To further confirm whether IAPP regulated the function of CD8^+^T cells through copper, we combined copper chelator ammonium tetrathiomolybdate to detect its effect on CD8^+^T cells viability and function with CuCl_2_ and elesclomol, the cell viability and function of CD8^+^T cells were increased after downregulation of IAPP (**Figures 5G–J**, *P*<0.01, *P*<0.001). Copper carrier induced cell death relies on mitochondrial respiration (21), so we detected the viability of CD8^+^T cells and the expression of GzmB, TNF-α and IFN-γ with CuCl_2_ and rotenone, the cell viability and function of CD8^+^T cells were increased after downregulation of IAPP (**Supplementary Figure S6**, *P*<0.01, *P*<0.001). To further explore the effect of IAPP on CD8^+^T cells, we overexpressed IAPP in CD8^+^T cells, and found that IAPP increased copper concentration and inhibited the cell viability and secretion of cytokines (**Supplementary Figure S7**, *P*<0.001). The above results proved that mitochondrial respiratory regulated copper induced the viability and function of CD8^+^T cell, meaning downregulation of IAPP indeed enhanced CD8^+^T cells function by suppressing cuproptosis.

### Reducing cuproptosis enhances anti-BRCA function of CD8^+^T cells

**Figure 6:**
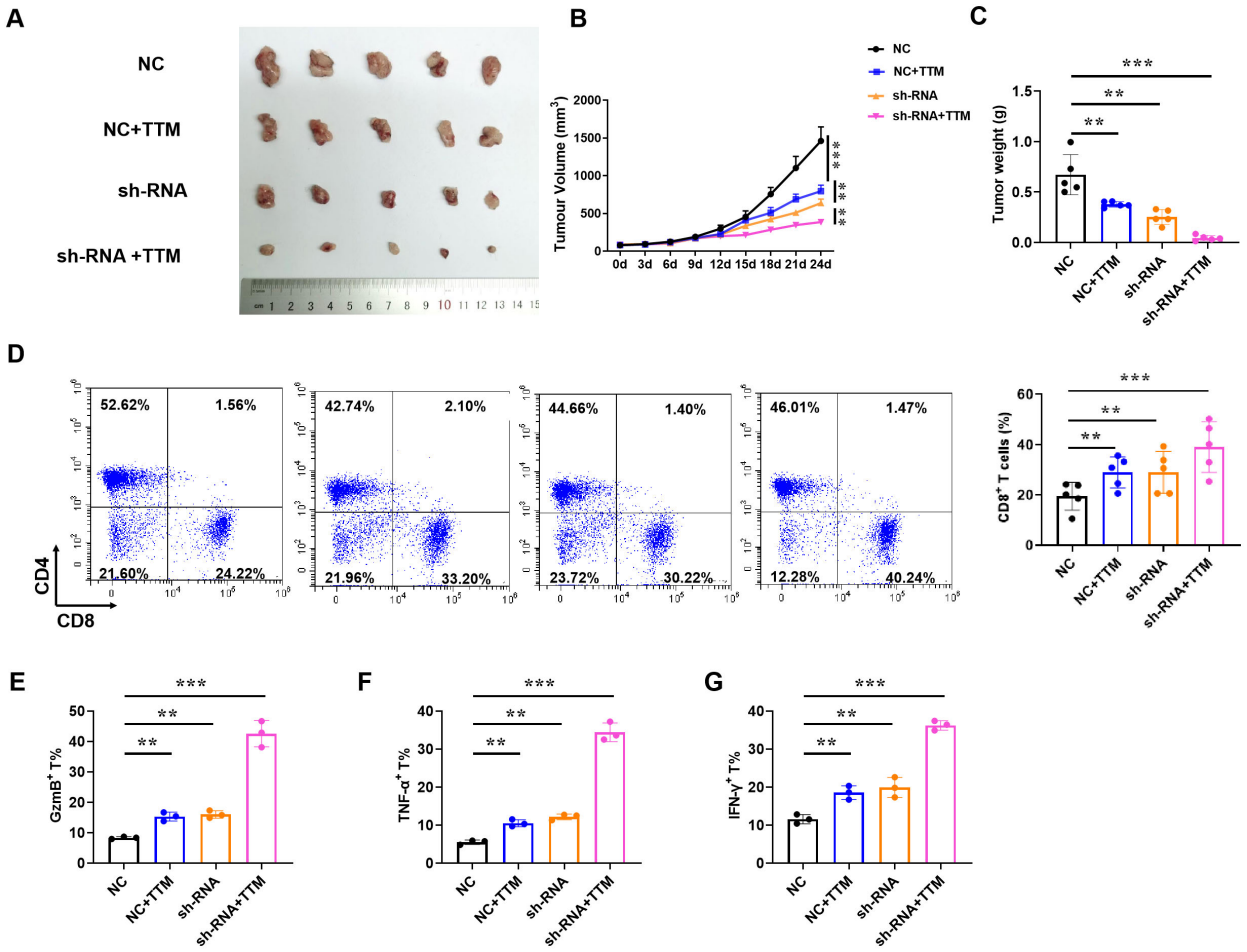
Reducing copper concentration induces anti-BRCA function of CD8^+^T cells. NY-ESO-1^+^ A375 cells were engrafted into NSG mice and NY-ESO-1-specific TCR T cells were injected via the tail vein. **(A–C)** Representative image of the tumor tissues, tumor volume, and tumor weight, respectively. **(D)**The proportion of CD4^+^ and CD8^+^ T cells in BRCA. **(E–G)** The proportion of GzmB^+^, TNF-α^+^, and IFN-γ^+^ in CD8^+^T cells (Mean ± SD; ^**^ *P*<0.01;^***^ *P*<0.001, n=5).

Since IAPP can mediate copper to affect the function of CD8^+^T cells *in vitro*, we then further proved its anti-BRCA function *in vivo*. Briefly, the mice loaded with tumors were treated with NC CD8^+^T cells, sh-RNA-IAPP (sh-RNA) CD8^+^T cells, and TTM, tumor size and immune cell profile was analyzed. Statistical analysis revealed that downregulation of IAPP notably suppressed BRCA growth, and its combination with TTM further enhanced the anti-tumor effect (**Figures 6A–C**, *P*<0.01, *P*<0.001). Flow cytometry analysis showed that proportion of CD8^+^T cells (**Figure 6D**) and expression of GzmB, TNF-α and IFNγ (**Figures 6E–G**, *P*<0.01, *P*<0.001) were significantly increased in sh-RNA+TTM group, indicating that downregulation of IAPP can reduce copper concentration and further promoted the anti-BRCA function of CD8^+^T cells *in vivo*.

### Downregulation of IAPP increases the anti-BRCA activity of Her2-CAR-T cells

**Figure 7:**
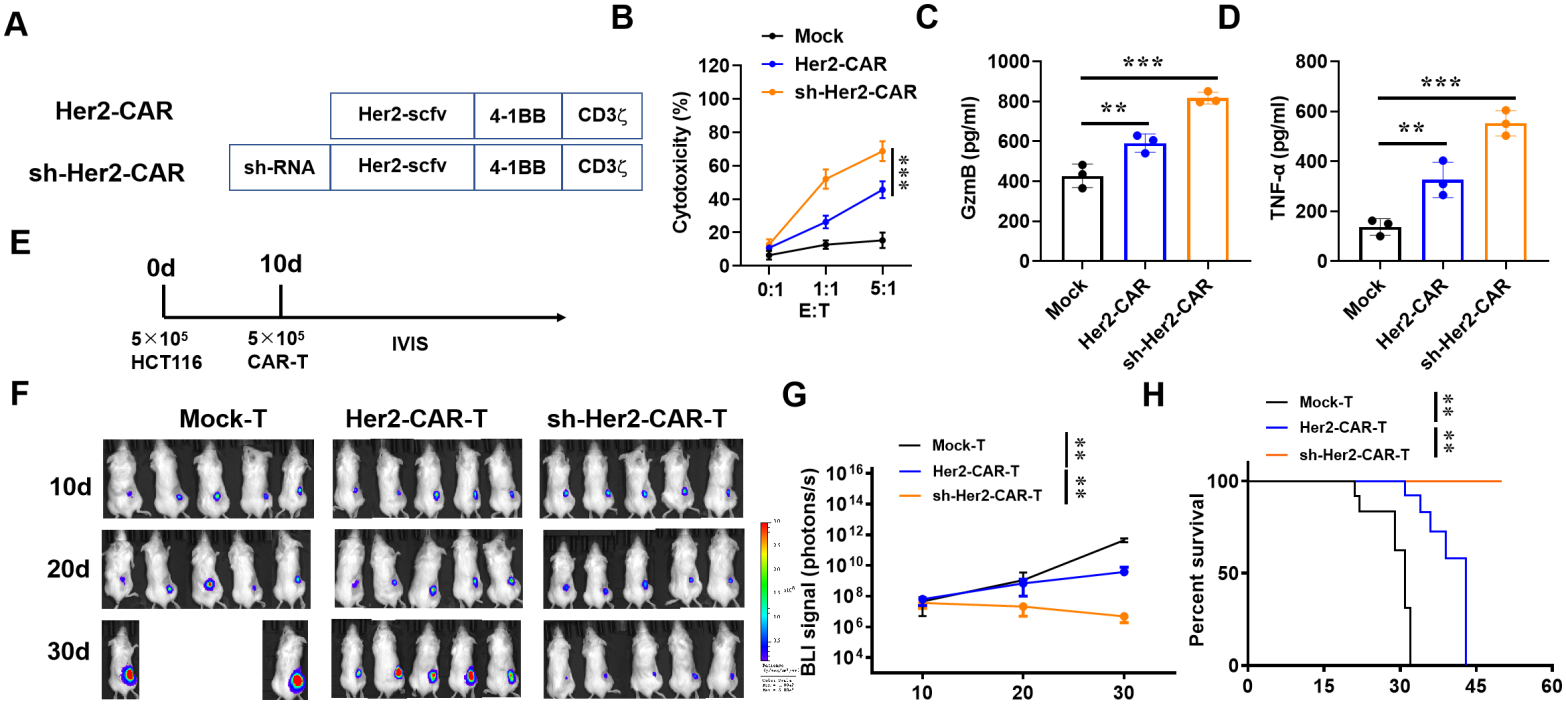
Downregulation of IAPP increases the anti-tumor activity of Her2-CAR-T cells. **(A)** Schematic representation of CARs. **(B)** Cytotoxicity analysis of CAR-T cells incubated with BT474 cells. **(C, D)** Production of GzmB and TNF-α in CAR-T cells after incubating with BT474 cells for 16 h. **(E)** 10^6^ MDA-MB-231 cells were injected into the right back of mice (n=5) for 10 d and infused with 5×10^6^ CAR-T cells (i.v.). **(F, G)** Bioluminescence of tumor and its statistics. **(H)** Kaplan-Meier survival curves (Mean ± SD; ^**^ *P*<0.01; ^***^ *P*<0.001).

To extend the application of IAPP, we constructed sh-IAPP into Her2-CAR to prepare sh-IAPP-Her2-CAR-T cells (**Figure 7A**). Mock, Her2-CAR-T and sh-IAPP-Her2-CAR-T cells were incubated with BT474 cells for 4 h at the ratio of 0:1, 1:1 and 5:1. Both Her2-CAR-T cells and sh-IAPP-Her2-CAR-T cells showed a higher cytotoxicity, while sh-IAPP-Her2-CAR-T cells were better than Her2-CAR-T cells (**Figure 7B**, *P*<0.001). Meanwhile, compared with Her2-CAR-T cells, sh-IAPP-Her2-CAR-T cells produced more GzmB, TNF-α and IFN-γ (**Figures 7C–E**, *P*<0.01, *P*<0.001). To further identify the data, we injected the MDA-MB-231 cells into NSG mice, and then treated with CAR-T cells at 10 days (**Figure 7F**). The final data demonstrated that sh-IAPP-Her2-CAR-T cells significantly restrained the tumor growth and prolonged survival *in vivo* (**Figures 7G–I**, *P*<0.01).

### Downregulation of IAPP promotes CD8^+^T viability and function through NF-κB pathway

**Figure 8:**
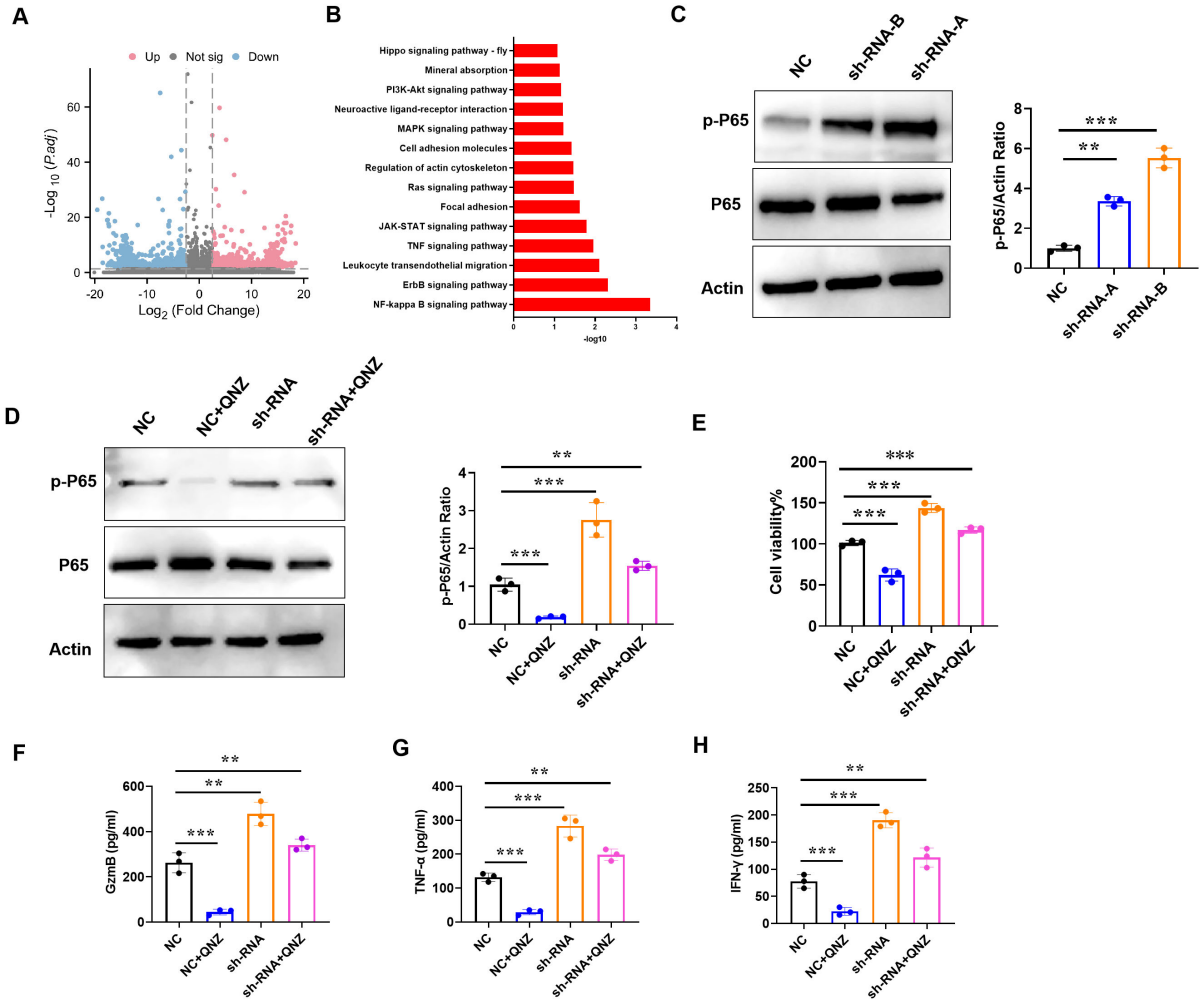
Downregulation of IAPP promotes viability and function of CD8^+^T cells through NF-κB pathway. **(A)** Volcano plots of DEGs in NC or sh-RNA groups. **(B)** KEGG analysis of DEGs enrichment in NC or sh-RNA groups. **(C)** Expression of p-P65 and P65 in CD8^+^T cells. **(D)** Expression of p-P65 and P65 in CD8^+^T cells treated QNZ. **(E)** Detection of CD8^+^T cells viability. **(F–H)** Expression of GzmB, TNF-α, and IFN-γ in CD8^+^T cells treated QNZ, respectively (Mean ± SD; ^**^ *P*<0.01; ^***^ *P*<0.001, n=5).

To explore the mechanism of IAPP, CD8^+^T cells transfected with NC and sh-RNA were subjected to RNA sequencing. The transcriptional profiles of sh-RNA CD8^+^T cells were largely different from those of NC CD8^+^T cells (**Figure 8A**). Through KEGG analysis, NF-κB signaling pathway was enriched in up-regulated different genes (**Figure 8B**). Studies have shown that activation of NF-κB pathway not only affects the development of T cells, but also promotes the proliferation and function of effector T cells (22, 23). To confirm whether NF-κB pathway was more active in sh-RNA CD8^+^T cells, p-P65 was detected using western blot, and results showed that downregulation of IAPP promoted the expression of p-P65 (**Figure 8C**, *P*<0.01 *P*<0.001). Additionally, CD8^+^T cell viability and expression of GzmB, TNF - α, and IFN - γ were restrained after adding NF-κB inhibitor QNZ (**Figure 8D-H**, *P*<0.01 *P*<0.001). All results proved that downregulation of IAPP promoted the functional marker protein expression of CD8^+^T cells through NF-κB pathway.

## Discussion

Current, cuprosis, a new mode of cellular demise that primarily found in cells that employ oxidative phosphorylation as their primary metabolic route for energy production (24–26) However, it has only been scarcely studied in relation to BRCA prognosis and treatment, as well as genes linked to copper-induced cell mortality. So, this study we uncovered 239 co-expressed immune cuprotosis-linked DEGs by analyzing BRCA differential genes and copper immunocytosis-related genes. And the prognostic risk model further offers novel insights into BRCA pathogenesis, potentially contributing to therapeutic and prognostic advancements. Notably, survival analysis and ROC curves robustly validated these ICR-Sig prognostic signatures, demonstrating a strong correlation with clinical observations, suggesting that ICRSig could serve as a potential risk predictor.

The immune (27, 28) and copper-related genes (29–31) have been confirmed to participate in tumor proliferation, differentiation, invasion and metastasis via regulating tumor progression and pathogenesis. Presently, various immune genes (32, 33) display differential expressions in breast cancer tissues, participating in numerous biological processes of breast cancer, such as tumor cell proliferation, apoptosis, and migration. In the tumor microenvironment, cancer cells and immune cells secrete a plethora of chemokines and cytokines, which modulate the occurrence and progression of tumors. Immune evasion is affected by PD-L1 upregulation, secretion of immunosuppressive cytokines (IL-6, IL-10, TGFβ, and MCSF) and suppressive immune cells recruitment such as M2 macrophages, regulatory T (Treg) cells and myeloid-derived suppressor cells (MDSCs), which eliminate immune-mediated tumor cell killing by suppressing Natural Killer (NK) cells and CD8^+^T cells (34). So finding the immune and copper-related genes is crucial for the diagnosis and treatment of tumors. Notably, in this study, the ESTIMATE Score was higher in low-risk patients than in high-risk patients. The risk score was positively correlated with the ICI Key Objective, which meant that the risk score might contribute to customized immunotherapy. Besides, the risk scoring model was negatively correlated with infiltration of cytotoxic immune cells, associated with tumor progression and immunosuppression. Therefore, the risk score could contribute to the prediction of the outcome of immunotherapy. Stem cells are regarded as an important cause of tumor recurrence and invasion (35). Our findings revealed that with a higher risk, cancer stem cells multiplied, contributing significantly to the poor prognosis observed in the high-risk group. However, the mechanism between the immune cuprotosis gene and immunity is still unclear. We conducted screening analysis suggests that IAPP may be a potential gene regulating CD8^+^T cells.

According to previous research, copper-triggered cell death is mediated by the ancient process of protein lipoylation. Surprisingly, only a small number of mammalian proteins are known to undergo lipoylation, and these are predominantly concentrated in the TCA cycle, where lipoylation is necessary for enzymatic function. Mitochondrial respiration has been shown to regulate copper ionophore-induced cell death (36–38). Our results have found that Our results found that downregulating IAPP in CD8^+^T cells does not affect their activity and function. In the presence of CuCl_2_, downregulation of IAPP promotes the vitality and function of CD8^+^T cells by mediating cuprotosis. Animal experiments combined with TTM further proved the anti-BRCA function of IAPP mediated CD8^+^T cells *in vivo*. The discovery of IAPP, a polypeptide comprising 37 amino acids and displaying roughly 50% homology with the neuropeptide calcitonin gene-related peptide (CGRP), was made through chemical analysis of the amyloid component in human pancreatic islet cell tumors. IAPP functions as a tumor marker for identifying pancreatic islet cell tumors. Qian Zhou et al. found that copper increased the levels of reactive oxygen species (ROS) in BV2 cells, which then activated NF-κB as an early survival (39). It is reported that NF-κB pathways are required for CD8^+^T cell-mediated killing effect (40, 41). Our sequencing results are enriched in NF-κB differential signaling pathways. Our results indicated that downregulation of IAPP mediated copper promotion of CD8^+^T cells viability and function through NF-κB pathway. Besides, given the downregulation of IAPP largely affected CD8^+^T cells function, so IAPP may acted as a new endogenous regulator of CD8^+^T cell biology, but this needed further to explored in the future. With the objective of advancing clinical implementation, we have engineered a Her2-CAR construct incorporating the downregulation of IAPP receptors. We found that down-regulating of IAPP receptor in Her2-CAR-T cells revealed higher cytotoxic activity against BRCA compared to Her2-CAR-T cells.

## Conclusions

Our experimental results also proved that IAPP is an immune cuprotosis gene and downregulation of IAPP mediated copper to promote the viability and function of CD8^+^T cells through NF-κB to play an anti-breast cancer role. IAPP was an ICR target for the diagnosis and treatment of breast cancer, which provides a theoretical basis for the treatment of breast cancer.

## Data Availability

The datasets presented in this study can be found in online repositories. The names of the repository/repositories and accession number(s) can be found below: https://www.ncbi.nlm.nih.gov/, PRJNA1119691.

## Supplementary material

Supplementary Figure 1 Kaplan-Meier survival curves of BRCA patients in different clinical subgroups. Age>65 **(A)**, Age<=65 **(B)** OS survival curves of high-risk and low-risk GC patients in subgroups of T1, T2, T3, T4, N0, N1, N2, N3, M0, and M1 **(C-L)**.

Supplementary Figure 2 Correlation analysis between other genes (GRIN2D, FN1, CXCL9, GSN, SCN3A, TFF1, NGB, SERPINA12, HSPB2, TH, KCNE1B, WT1, CACNA1H, CLEC6A, HBA1) and immune cells (Mean ± SD; ^**^ *P*<0.01;^***^ *P*<0.001).

Supplementary Figure 3 Western blot detection of downregulation of IAPP.

Supplementary Figure 4 Downregulation of IAPP does not affect the vitality and function of CD8^+^T cells without Cucl_2_ (Mean ± SD; ^**^ *P*<0.01;^***^ *P*<0.001).

Supplementary Figure 5 Downregulation of IAPP reduces cuprosis related proteins FDX1 and SLC31A1 in CD8^+^T cells (Mean ± SD; ^**^ *P*<0.01;^***^ *P*<0.001).

Supplementary Figure 6 Following the downregulation of IAPP, mitochondrial respiratory regulates the viability and function of CD8^+^T cells induced by copper. Detection of cell viability **(A)** and GzmB **(B)**, TNF-α **(C)**, IFN-γ **(D)** secretion by CD8^+^T cells with or without 0.1µM Rot and 1µM Cucl_2_ (Mean ± SD; ^**^ *P*<0.01; ^***^ *P*<0.001).

Supplementary Figure 7 Upregulation of IAPP mediates copper to inhibited CD8^+^T cell viability and function. **(A)** Western blot detection of expression of IAPP. **(B)** Detection of the mRNA expression. **(C)** Detection of copper concentration in IAPP CD8^+^T cells by ICP-MS. Detecting the vitality of CD8^+^T cells and the expression of GzmB **(E)**, TNF-α **(F)**, IFN-γ **(G)** after upregulation of IAPP. Detection of cell viability **(H)** and GzmB **(I)**, TNF-α **(J)**, IFN-γ **(K)** secretion by CD8^+^T cells with or without TTM (Mean ± SD; ^**^ *P*<0.01; ^***^ *P*<0.001).
