# Supplementary figures and images for: IAPP blocks anti-breast cancer function of CD8+T cells via targeting cuproptosis
Source: Front Immunol. 2024 Nov 25;15:1481129. doi: 10.3389/fimmu.2024.1481129 (PMC11625781; doi:10.3389/fimmu.2024.1481129)

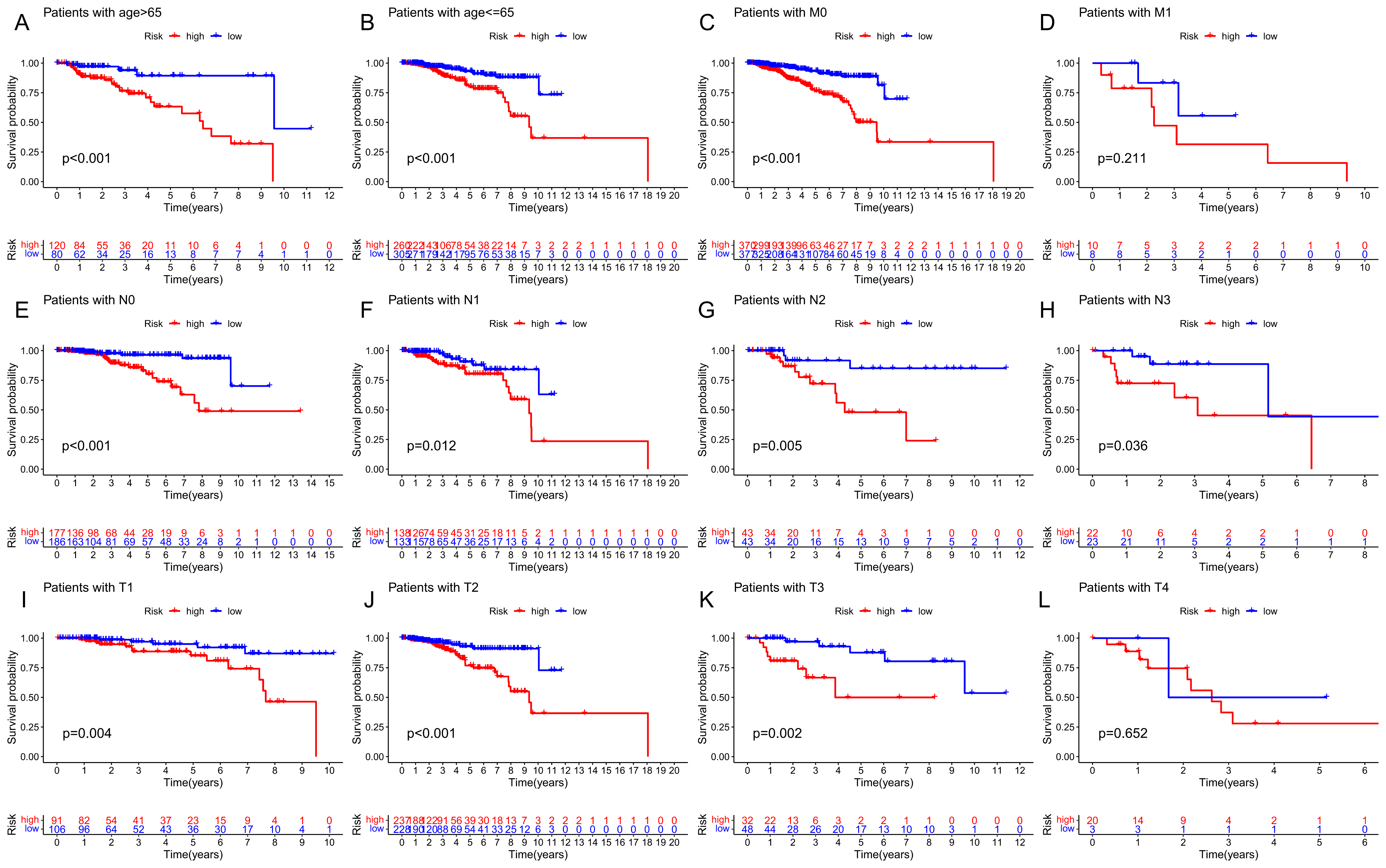

Supplement: Supplementary Figure 1 — Kaplan-Meier survival curves of BRCA patients in different clinical subgroups. Age>65 (A), Age<=65 (B) OS survival curves of high-risk and low-risk GC patients in subgroups of T1, T2, T3, T4, N0, N1, N2, N3, M0, and M1 (C-L). [file Image1.tif]

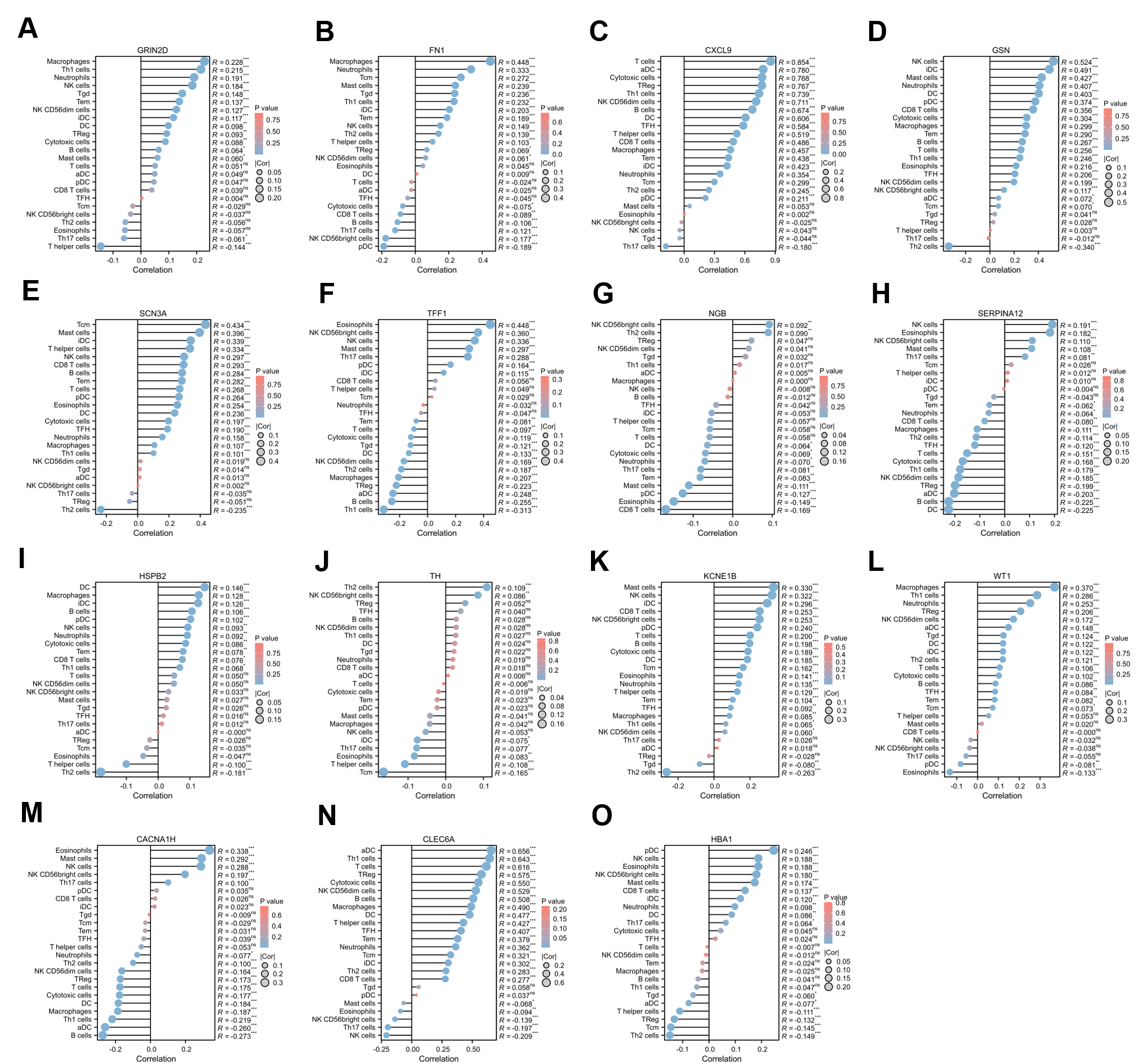

Supplement: Supplementary Figure 2 — Correlation analysis between other genes (GRIN2D, FN1, CXCL9, GSN, SCN3A, TFF1, NGB, SERPINA12, HSPB2, TH, KCNE1B, WT1, CACNA1H, CLEC6A, HBA1) and immune cells (Mean ± SD; ** P<0.01;*** P<0.001). [file Image2.tif]

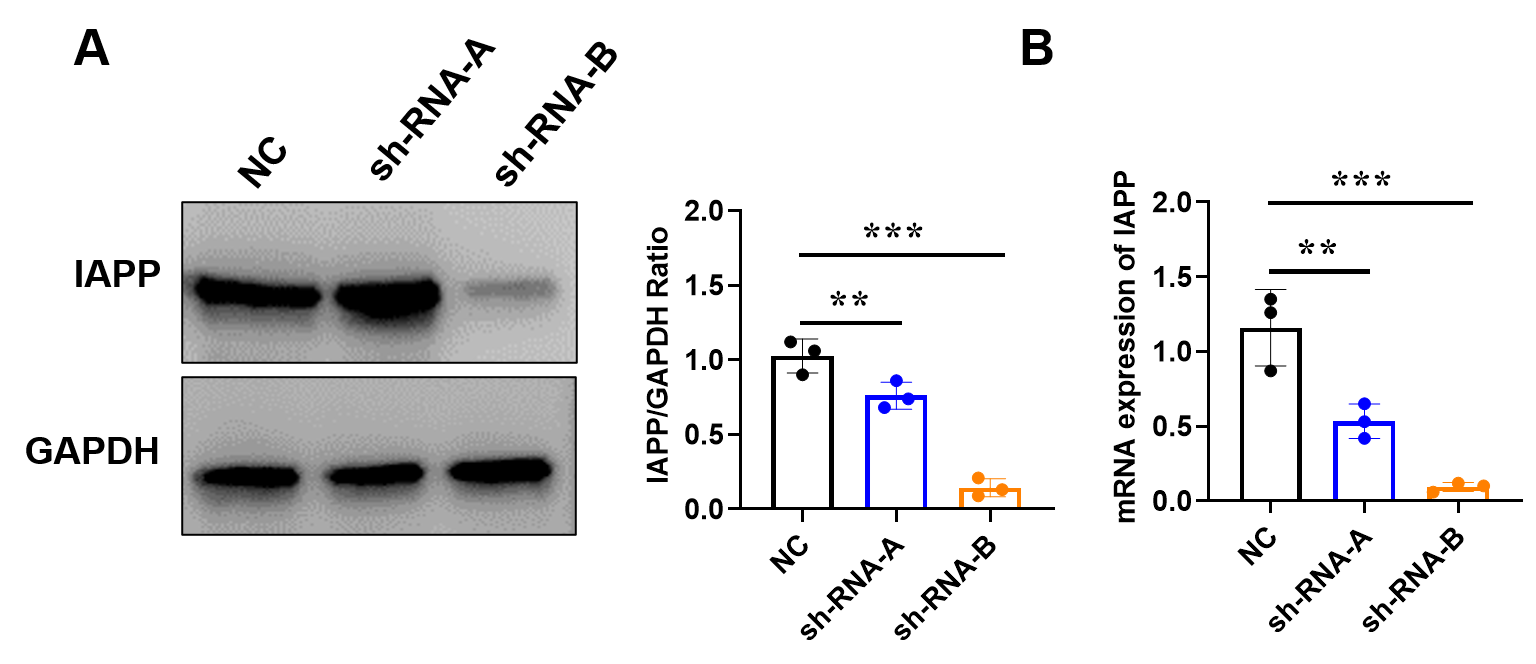

Supplement: Supplementary Figure 3 — Western blot detection of downregulation of IAPP. [file Image3.tif]

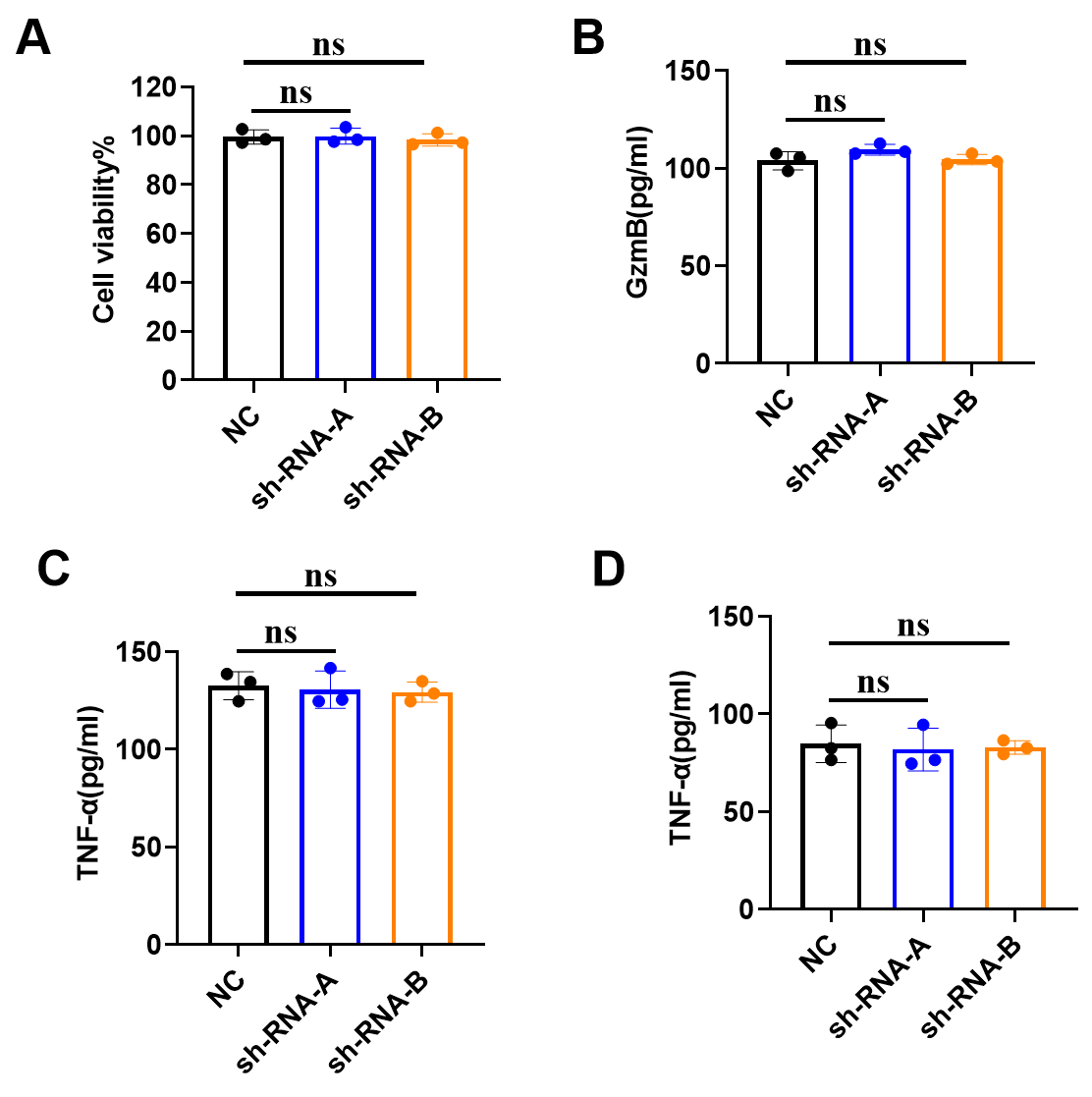

Supplement: Supplementary Figure 4 — Downregulation of IAPP does not affect the vitality and function of CD8+T cells without Cucl2 (Mean ± SD; ** P<0.01;*** P<0.001). [file Image4.tif]

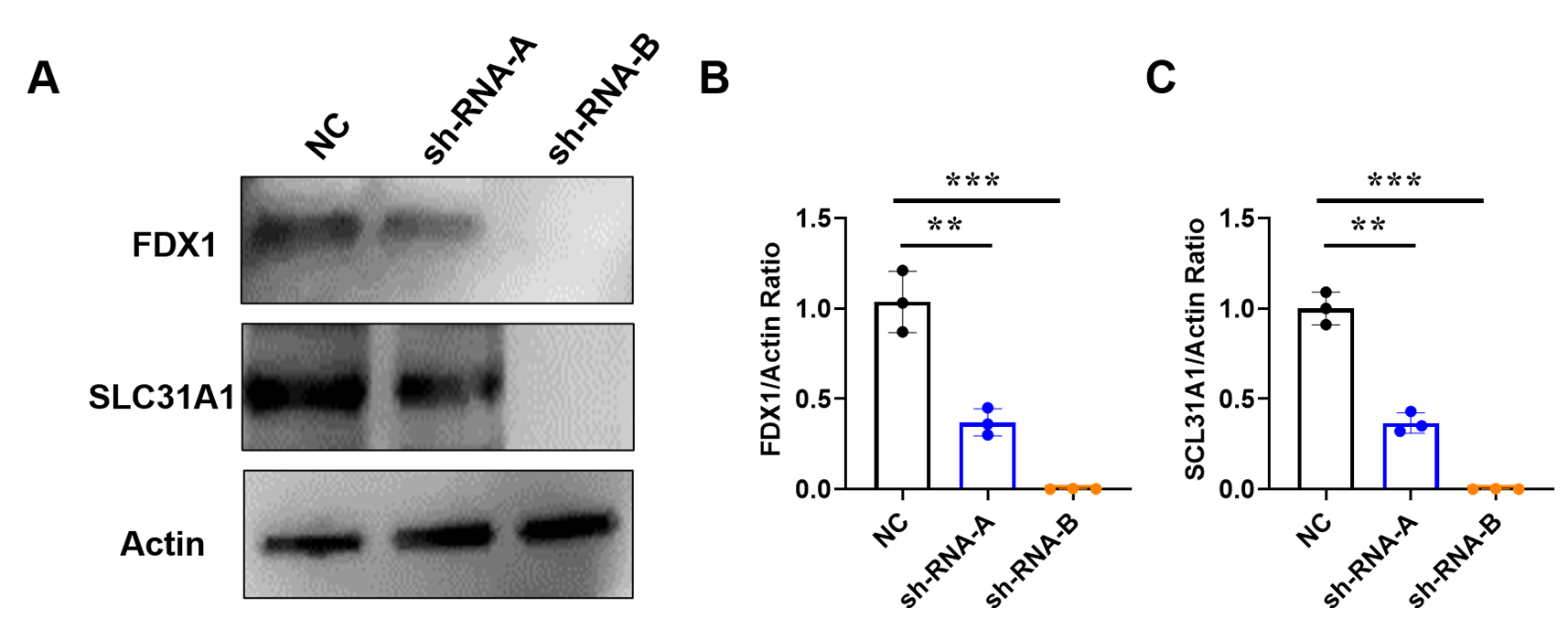

Supplement: Supplementary Figure 5 — Downregulation of IAPP reduces cuprosis related proteins FDX1 and SLC31A1 in CD8+T cells (Mean ± SD; ** P<0.01;*** P<0.001). [file Image5.tif]

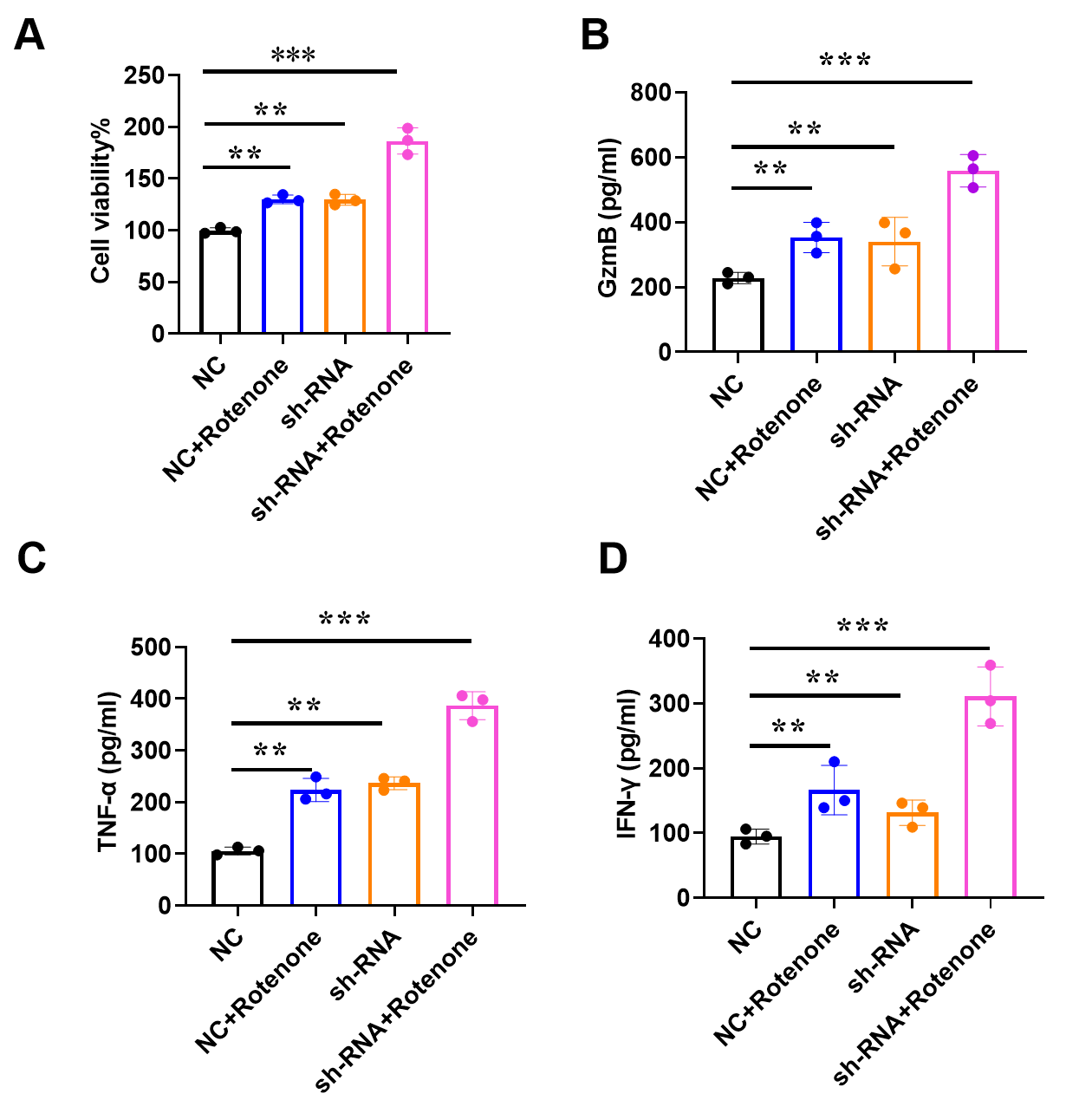

Supplement: Supplementary Figure 6 — Following the downregulation of IAPP, mitochondrial respiratory regulates the viability and function of CD8+T cells induced by copper. Detection of cell viability (A) and GzmB (B), TNF-α (C), IFN-γ (D) secretion by CD8+T cells with or without 0.1µM Rot and 1µM Cucl2 (Mean ± SD; ** P<0.01; *** P<0.001). [file Image6.tif]

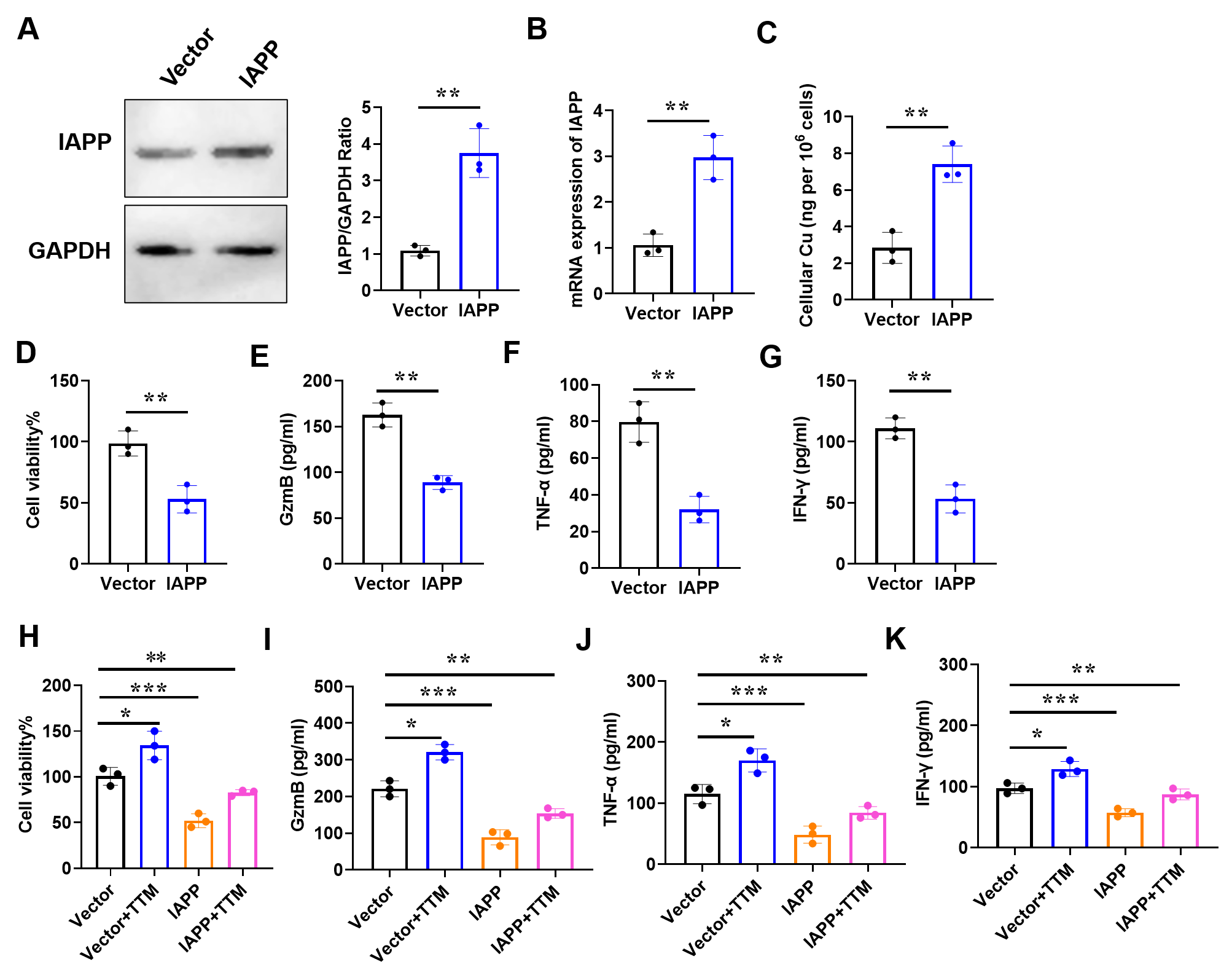

Supplement: Supplementary Figure 7 — Upregulation of IAPP mediates copper to inhibited CD8+T cell viability and function. (A) Western blot detection of expression of IAPP. (B) Detection of the mRNA expression. (C) Detection of copper concentration in IAPP CD8+T cells by ICP-MS. Detecting the vitality of CD8+T cells and the expression of GzmB (E), TNF-α (F), IFN-γ (G) after upregulation of IAPP. Detection of cell viability (H) and GzmB (I), TNF-α (J), IFN-γ (K) secretion by CD8+T cells with or without TTM (Mean ± SD; ** P<0.01; *** P<0.001). [file Image7.tif]
